# The Mechanics of Surgery

**Published:** 1899-12

**Authors:** 


THE MECHANICS OF SURGERY. Comprising detailed descriptions, illustrations and lists of instruments, appliances and furniture necessary in modern surgical art. By CHARLES TRUAX. Chicago. 1899.

As a rule when one picks up a book of this character the first thought is one of disinterested curiosity, for the idea is so intimately associated with uninteresting and slap-dash illustrations of individual manufacturing catalogues of surgical instruments. In the case of the mechanics of surgery, however, Mr. Truax has prepared a volume which is in no sense a catalogue. It has no price lists, is not confined to any one make of instrument, nor does it contain the slightest suspicion of individual advertisement. These virtues make it distinctly a work of art, and a most valuable educational volume. How little the average practitioner knows of the various instruments used in medicine and surgery and their uses is well known; so well known that it needs no more than passing reference here. Certain it is that such a work has long been needed. True, there have been publications of volumes illustrative of the uses of instruments, but they have been cursed with the individuality of the publisher, who in every case has been a maker of instruments and who confined his literary and artistic efforts to booming his own make. This is not so with the Truax volume. No matter where an instrument is made; no matter who the maker, if it is valuable it has a place in the Truax book.

There is a lamentable lack of attention paid to instruments and their proper care and uses in the text-books on surgery; if one needs an instrument and goes to a dealer, he is limited in his choice to the dealer's stock, which too often is limited, either in quantity, quality or make. If he appeals to a catalogue he is confronted with a lot of wood-cut abortions, trimmed with the glossy language of commercial self-laudation.

In the Truax book all this is obviated. It was prepared to fill an unoccupied nook in surgical literature and it comes pretty near meeting all the requirements. Mr. Truax is an instrument maker. It is not his business; it is his profession; yet he does not confine himself to his own make of appliances, but illustrates the most serviceable and best instruments made, whether they are manufactured by Truax, Tieman, Kny, or any of the lesser lights. And strangely enough he confines himself to instruments in contradistinction to previous workers in this field who, after describing their own instruments, proceed to give voluntary advice regarding operative methods, which judging by the general aspects is more or less of a rehash of surgical text-books, prepared by medico-literary hacks.

Mr. Truax's experience has been gained by close attendance at clinics, both in this country and Europe, and by personal work in the preparation of the articles necessary to the physician and surgeon; and while he tells the uses of an instrument he does not presume to direct its use. For instance, he explains how a plaster-of-Paris bandage is prepared, but not how it is applied. He leaves something for the colleges to teach.

One very gratifying portion of the book is that which deals with the standard nomenclature of instruments. A plea is made for uniformity. A periosteal elevator is the name of an instrument which is commonly and variously called a levator, raspatory, dry dissector, elevator, periosteatome. Spring dressing forceps are spring dressing forceps and not thumb forceps, dissecting forceps, plain artery forceps, tissue forceps, or nippers. These are illustrations of evils Mr. Truax is endeavoring to correct.

A chapter is devoted to the history of surgical instruments, which is highly interesting; another is given to the construction and care of instruments, which is valuable, coming as it does from a man who knows all about them from the forge to the surgeon's hands. Then follows careful description and admirable illustrations of everything pertaining to instrumental medicine and surgery in general and special and regional surgery.

The book is a complete work and it is valuable. And that is a good deal to say of any work of literature in these days.

